# TRIM6 promotes colorectal cancer cells proliferation and response to thiostrepton by TIS21/FoxM1

**DOI:** 10.1186/s13046-019-1504-5

**Published:** 2020-01-28

**Authors:** Shuier Zheng, Chenliang Zhou, Yonggang Wang, Hongtao Li, Yong Sun, Zan Shen

**Affiliations:** Department of Oncology, Shanghai Jiao Tong University Affiliated Sixth People’s Hospital, 600 Yishan Road, Shanghai, 200233 China

**Keywords:** FoxM1, Cell cycle arrest, TIS21, TRIM6

## Abstract

**Background:**

Tripartite motif-containing proteins (TRIM) play a crucial role in carcinogenesis. Little attention has been focused on the possible functions of TRIM6 on carcinogenesis.

**Methods:**

The expression levels of TRIM6 were assessed in colorectal cancer (CRC) samples. TRIM6 expression was knocked down in CRC cell lines, and subjected to Cell counting kit-8 (CCK-8), bromodeoxyuridine (BrdU) incorporation and cell cycle assays. Immunoprecipitation and proteomics analysis was performed to identify potential associated proteins of TRIM6.

**Results:**

TRIM6 expression was up-regulated in CRC samples and TRIM6 expression may be an independent prognostic marker for CRC. Knocking down TRIM6 expression suppressed CRC cell proliferation, induced cell cycle arrested at G2/M phase and increased sensitivity to 5-fluorouracil and oxaliplatin. TIS21, an anti-proliferative protein involved in the regulation of G2/M arrest, was identified as an interaction partner of TRIM6. Moreover, CRC cells with TRIM6 overexpression showed decreased TIS21 protein stability. TIS21 ubiquitination was increased in CRC cells overexpressing TRIM6, but not in those overexpressing TRIM6 E3 catalytic mutant (C15A). Further, Lys5 was essential for TRIM6 mediated TIS21 ubiquitination. TIS21 overexpression reversed the induced effects of TRIM6 overexpression on CRC cell proliferation, and the levels of forkhead box M1 (FoxM1), phosphorylated FoxM1, Cyclin B1 and c-Myc. Thiostrepton, a specific inhibitor for FoxM1, was less effective in anti-proliferative activity against CRC cells with lower level of TRIM6 in vitro and in vivo.

**Conclusions:**

Our study suggests that TRIM6 promotes the progression of CRC via TIS21/FoxM1.

## Background

Colorectal cancer (CRC) is the third most common cancer in men and the second in women throughout the world. More than 700,000 patients died from CRC annually, thus making CRC the fourth most common cause of cancer-related death [1]. CRC will account for more than 1.1 million deaths and 2.2 million new diagnosed cases per year worldwide by 2030 [2]. It is highly desirable to identify precise biomarkers, which will help the diagnosis and treatment of CRC, and further facilitate the prediction or monitoring of cancer recurrence.

Tripartite motif-containing proteins (TRIM), containing more than 70 members, play critical roles in immune responses, carcinogenesis and chemoresistance [3–5]. Tripartite motif-containing protein 6 (TRIM6) is a member of TRIM family proteins. *TRIM6* gene locates to chromosome 11p15, where it resides within a TRIM gene cluster that includes TRIM5, TRIM21, TRIM22, TRIM34, and a TRIM pseudogene [6]. Like other TRIM family proteins, TRIM6 has a tripartite motif and possesses E3-ubiquitin ligase activity [7]. Previous studies have revealed the roles of TRIM6 in viral infection and inflamation responses. Rajsbaum et al. reported that TRIM6 can active IκB kinase-ε (IKKε) and promote the induction of downstream type I interferon (IFN-I) stimulated genes (ISGs), thus facilitating viral control [8]. The pathogenic Nipah virus (family *Paramyxoviridae*) can inhibit IKKε signaling via targeting the degradation of TRIM6 [9], which further demonstrated the antiviral responses of TRIM6. On the contrary, another study reported that TRIM6 can enhance Ebola virus replication by promoting the ubiquitination of an important viral protein VP35 [7]. However, little attention has been focused on the possible functions of TRIM6 on carcinogenesis.

In the present study, we reported that TRIM6 expression was significantly elevated in CRC samples and explored the correlation between TRIM6 expression and clinical parameters of CRC patients. Knocking down TRIM6 expression suppressed CRC cell proliferation and induced cell cycle arrested at G2/M phase. Mechanistically, we used immunoprecipitation followed by proteomics analysis to explore potential interaction proteins affecting TRIM6 functions. TIS21, an antiproliferative protein involved in the regulation of G2/M arrest [10], was identified as an interaction partner of TRIM6. Our study has revealed the clinical significance of TRIM6 in the progression of CRC and may provide a novel therapy target for CRC patients.

## Materials and methods

### CRC tissue samples

This study was approved by the Institutional Review Board of Shanghai Jiao Tong University Affiliated Sixth People’s Hospital. Two cohorts of patients treated at Shanghai Jiao Tong University Affiliated Sixth People’s Hospital were enrolled in this study after written consent informed was collected. Cohort 1 included 35 CRC patients treated between 2016 and 2017, and 35 pairs of fresh CRC specimens and their adjacent mucosa tissues were obtained from these patients, and stored at − 80 °C until analysis. Cohort 2 contained 90 CRC patients treated between 2010 and 2012 with clinical information and prognosis information (Table 1), and paraffin-embedded CRC specimens were available for immunohistochemical (IHC) staining.

**Table 1:** Clinicopathological characteristics and TRIM6 expression (*n* = 90)

Characteristic	Cases	%
Gender		
Male	48	53.3
Female	42	46.7
Age (years)		
≥ 65	29	32.2
< 65	61	67.8
Tumor size (cm)		
≥ 4.0	60	66.7
< 4.0	30	33.3
Pathologic differentiation		
Well/Moderate	75	83.3
Poor	15	16.7
Clinical stage		
I/II	43	47.8
III	47	52.2
Metastasis		
yes	17	18.9
no	63	81.1
Vital status (at followed-up)		
Alive	42	46.7
Dead	48	53.3
CEA levels (ng/mL)		
≥ 3.5	58	64.4
< 3.5	32	35.6
TRIM24 expression		
Low	34	37.8
High	56	62.2

### Cell lines

Human normal colorectal mucosa cell line, FHC, and CRC cell lines, LOVO, Sw620, HCT-8 and HCT116, were purchased from Cell Bank of Shanghai Institute of Cell Biology, Chinese Academy of Sciences, and grown in a 37 °C incubator at 5% CO2 using DMEM medium (Hyclone, Logan, UT, USA) containing 10% fetal bovine serum (Life Technology, Grand Island, NY, USA).

### Quantitative RT-PCR (qRT-PCR)

Total RNA was extracted from tissues with Trizol reagent (Life Technology). One μg RNA was processed to cDNA synthesis with the reverse transcription kit (Fermentas, Hanover, MD, USA). Real-time PCR reactions were performed in triplicates with SYBR Green mix (Thermo Fisher Scientific (Rockford, IL, USA) on ABI-7300 system (Applied Biosystem, Foster City, CA) according to the manufacturer’s instructions. The primer sequences were listed in Additional file 1: Table S1. The relative mRNA levels were normalized to that of GAPDH.

### Immunohistochemical staining

Paraffin-embedded CRC specimens were cut at 4 μm, and deparaffinizaion and rehydration was performed with xylene, a solution of xylene and ethanol and a series ethanol solutions. Antigen retrieval was carried out by boiling slides in 0.01 M sodium citrate buffer (pH 6) at 100 °C for 15–20 min. Subsequently, the slides were block with 3% hydrogen peroxide for 30 min and then with 5% bovine serum albumin (BSA) for 2 h at room temperature (RT). After incubation with anti-TRIM6 (Bioss Inc., Woburn, Massachusetts, USA), anti-TIS21 (Abcam, Cambridge, MA, USA), anti-p-FoxM1(Thr600) (Affinity, Cincinnati, OH, USA), anti-p-FoxM1(Ser35) (Affinity) overnight at 4 °C, the slides were incubated with horseradish peroxidase (HRP)-conjugated secondary antibodies (Longislandbio, Shanghai, China) for 1 h at RT, developed with a DAB staining kit (Longislandbio) and counterstained with hematoxylin.

### Western blotting

Cells or tissues were lysed in RIPA buffer containing protease inhibitor cocktail (Beyotime Biotech., Shanghai, China) on ice for 30 min, and the lysates were collected by centrifugation. Equal amounts of protein were mixed with lamini loading buffer, boiled for 5 min, separated by 10% or 15% sodium dodecyl sulphate/polyacrylamide gel electrophoresis, and transferred to nitrocellulose membranes. The membrane was blocked in 5% non-fat milk for 1 h at room temperature, and then incubated with the primary antibody, anti-TRIM6 (1:1000, Proteintech, Chicago, IL, USA), anti-TIS21 (1:200, Abcam), anti-cleaved-caspase3 (1:1000, Abcam), anti-Cyclin B1 (1:500, Cell Signaling Technology, Danvers, MA, USA), anti-c-Myc (1:1000, Abcam), anti-forkhead box M1 (FoxM1) (1:1000, Cell Signaling Technology), anti-p-FoxM1(Thr600) (1:1000, Cell Signaling Technology), anti-p-FoxM1(Ser35) (1:1000, Cell Signaling Technology), and anti-GAPDH (1:2000, Cell Signaling Technology) overnight at 4 °C. After incubation with HRP-conjugated secondary antibodies (Beyotime Biotech.), the signal was detected using an ECL kit (Pierce, Rockford, IL, USA).

### Construction of plasmids

TRIM6, TRIM6 with substitute mutation of C15A and FoxM1 cDNA was cloned into the pcDNA3.1-myc vector (Life Technology) to express Myc-tagged TRIM6, TRIM6 E3 catalytic mutant (C15A) and FoxM1, respectively. The coding sequence for TRIM6, wild-type TIS21 (WT) or TIS21 with substitute mutations of K5R, K51R or K150R was cloned into the pCMV-Tag2 plasmid (Stratagene, La Jolla, CA, USA) to expressed FLAG-tagged TRIM6, WT and mutant TIS21. TRIM6 and FoxM1 cDNA was cloned into pGEX-2 T vector to produce GST fusion protein. The plasmids were verified by double enzyme digestion and DNA sequencing.

### Construction of shRNAs targeted TRIM6

shRNAs specially targeting TRIM6 (shTRIM6–1, shTRIM6–2 and shTRIM6–3) were designed and synthesized as listed in Additional file 1: Table S2. Scrambled shRNA sequence was also generated as negative control (shNC). After annealing, the double-stranded DNA was ligated into AgeI/EcoRI digested pLKO.1 plasmid (Addgene, Cambridge, MA, USA) and verified by DNA sequencing. Lentivirus was produced by transfection of plasmids into 293 T cells with Lipofectamine 2000 (Life Technology) following the manufacture’s instruction as previously described [11].

### Cell counting kit-8 (CCK-8) and bromodeoxyuridine (BrdU) incorporation assays

CCK-8 and BrdU incorporation assays were performed to analyze cell proliferation. Cells were plated into 96-well plate at a density of 3000 per well and cultured at 37 °C overnight. The cells were infected with TRIM6 shRNA/shNC, or transfected with pcDNA3.1-myc-TIRM6/ pCMV-Tag2-TIS21 as indicated in the figure legends, and cultured for 0 h, 24 h, 48 h and 72 h. For CCK-8 assay, the medium was replaced by DMEM medium containing 10% CCK-8 solution (SAB biotech. College Park, MD, USA and cultured at 37 °C for 1 h. Absorbance at 450 nm was determined using a microplate reader.

BrdU Cell Proliferation ELISA Kit (Abcam) was used for BrdU incorporation assay. In brief, cultures were labeled with BrdU for 2 h, incubated with fixing solution and then with mouse anti-BrdU for 1 h. After incubation with HRP-conjugated goat anti-mouse antibody, the cells were stained with the peroxidase substrate. Quantification of BrdU-labeled cells was measured using a microplate reader.

### Cell cycle analysis

Cells were plated in 6-well plates and treated as indicated in the figure legends. After 48 h, the cells were collected, fixed with ice-cold ethanol at 4 °C overnight, and labelled with propidium iodine (PI, Sigma-Aldrich). Cell cycle was analyzed with flow cytometry (BD Biosciences, Franklin Lakes, NJ, USA) according to the manufacturer’s instructions.

### Chemosensitivity assay

To test whether TRIM6 influences the IC50 (drug concentration producing 50% growth inhibition) of 5-fluorouracil (5-FU) and oxaliplatin (L-OHP), HCT-8 and HCT116 were infected with TRIM6 shRNA/shNC, and treated with 300, 400, 500 or 600 μM of 5-FU (Xdhelp, Shanghai, China) or 40, 60, 80 or 100 μM of L-OHP (Sanofi Aventis, Shanghai, China). After 24 h of culture, CCK-8 assay was performed to calculate the IC50.

To test the effect of TRIM6 on L-OHP, 5-FU-induced apoptosis, HCT-8 and HCT116 were infected with TRIM6 shRNA/shNC, and treated with 400 μM 5-FU (Xdhelp, Shanghai, China), 64 μM L-OHP (Sanofi Aventis, Shanghai, China) or vehicle (DMSO) for 24 h. The cells were collected and stained with Annxin V/PI (KeyGEN Biotech, Nanjing, China). Cell apoptosis was assessed by flow cytometry.

### Primary CRC cell isolation and treatment

Primary CRC cells were isolated from 12 patients who were admitted to Shanghai Jiao Tong University Affiliated Sixth People’s Hospital as previously described [12] after written informed consent was obtained. TRIM6 expression was determined by qRT-PCR. The cells seeded on 96-well plates were exposed to 400 μM 5-FU, 64 μML-OHP, 2 μM Thiostrepton (TST, Sigma-Aldrich, St. Louis, MO, USA) or vehicle (DMSO) for 48 h. CCK-8 assay was performed as mentioned above and the percentage of proliferation inhibition was calculated with the following formula: Inhibition rate (%) = (OD_vehicle_– OD_treatment_)/OD_vehicle_.

### Immunoprecipitation (IP) and liquid chromatography/mass spectrometry (LC/MS) analysis

pCMV-Tag2-TIRM6 or pCMV-Tag2 vector was transfected into 293 T cells, and 48 h later, 293 T cells were harvested and extracted in RIPA buffer. The overexpression of FLAG-TRIM6 was confirmed by western blotting. Following pre-cleared with IgG and protein A/G beads (Santa Cruz Biotechnology, Santa Cruz, CA, USA) at 4 °C for 2 h, extracts were incubated with anti-FLAG beads (Sigma-Aldrich) overnight at 4 °C. The immunoprecipitated protein complexes were eluted with FLAG peptide (Sigma-Aldrich), resolved on SDS-PAGE, and stained with Coomassie Brilliant Blue. Several differential bands were excise, digested with trypsin, and analyzed by LC/MS.

### Co-IP experiments

Cell lysates prepared from HCT-8 and HCT116 cells with RIPA buffer were incubated with anti-TRIM6 (Bioss Inc.), anti-TIS21 (Santa Cruz Biotechnology) or control IgG (Santa Cruz Biotechnology) for 2 h at 4 °C and then with protein A/G Plus agarose (Santa Cruz Biotech.) for 2 h at 4 °C. The immunoprecipitated proteins were analyzed by western blotting analysis.

### GST-pull down assay

GST fusion proteins of TRIM6 and TIS21, and GST protein were produced in *Escherichia coli* and conjugated to glutathione 4B beads (GE Healthcare, Pittsburgh, PA, USA). HCT-8 cell lysate was incubated with GST fusion proteins or GST protein for 2 h at 4 °C. The beads were washed three times with RIPA buffer, boiled with SDS sample buffer, and analyzed by western blotting.

### Half-life of TIS21

HCT-8 cells were transfected with pcDNA3.1-myc-TIRM6 or pcDNA3.1-myc (Vector) for 24 h, and exposed to 20 mM cycloheximide (CHX, Sigma-Aldrich). Cell lysate was prepared at 0, 3 and 6 h after exposure and subjected to western blotting analysis.

### Ubiquitination analysis

Cell lysates prepared from HCT-8 cells transfected with pcDNA3.1-myc-TIRM6 or pcDNA3.1-myc-TRIM6 (C15A) were reacted with anti-TIS21 or control IgG. The immunoprecipitated complexes were subjected to western blotting analysis using anti-ubiquitin (Abcam).

The 293 T cells were transfected with plasmids expressing myc-TRIM6, His-ubiquitin and FLAG-TIS21 (WT, K5R, K51R or K150R). Two days later, cells were harvested and sonicated in buffer A (20 mM imidazole, 5 M guanidine-HCl, 100 mM Na2HPO4/NaH2PO4, pH 8.0). Cell lysates were incubated with nickelnitrilotriacetic acid beads (Qiagen) at room temperature for 1 h. The beads were washed three times with buffer A, twice with buffer B (20 mM imidazole, 1 M guanidine-HCl, 100 mM Na2HPO4/NaH2PO4, pH 8.0), and then twice with buffer C (20 mM imidazole, 25 mM Tris, pH 6.5). The immunoprecipitated proteins were analyzed by western blotting analysis with anti-FLAG (Abcam).

### Immunofluorescence

HCT-8 or HCT116 cells cultured on the coverslips were washed twice in phosphate-buffered saline (PBS), fixed in 4% paraformaldehyde for 30 min, and then blocked with 5% BSA at RT for 1 h. The cells were incubated with rabbit anti-TRIM6 (Bioss Inc.) and mouse anti-TIS21 (Novus Biologicals, Inc.; Littleton, CO, USA) overnight at 4 °C. Cells were washed three times with PBS, and then incubated with the Alexa Fluor 555-labeled goat anti-rabbit IgG(H+L) (Beyotime Biotech.) and Alexa Fluor 488-labeled goat anti-mouse IgG(H + L) (Beyotime Biotech.) at room temperature for 1 h. After washing thrice with PBS, 4′-6-diamidino-2-phenylindole (DAPI, Beyotime Biotech.) was used to stain nuclei.

### In vivo tumorigenicity assay

All procedures were approved by Animal Care and Use Committee, Shanghai Jiao Tong University Affiliated Sixth People’s Hospital. Male nude mice (4–6 weeks old) were housed under specific pathogen-free conditions. Cell suspensions of HCT-8 expressing shNC or shTRIM6 cells (5 × 10^6^) were injected subcutaneously into the nude mice (6 mice for each group, randomly assigned). On the 33th day after inoculation, the tumors were resected, photographed and weighed.

A xenograft model was established to evaluate the outcome of TST treatment. Nude mice (34 mice for each cell line, randomly assigned) were subcutaneously injected with HCT116 or SW620 cells (5 × 10^6^ cells per mouse). On the 12th day after inoculation, the mice were randomly divided into two groups (*n* = 17 per group), and administrated with TST (500 mg/ kg /day) or vehicle by intraperitoneal injection every three days. On the 33th day after transplantation, 5 mice of each group were sacrificed and xenografts were weighed. Overall survival analysis was performed on the remaining mice (*n* = 12 per group).

### Statistical analysis

Statistical analysis was performed using GraphPad Prism 6 software (San Diego, CA, USA). Statistically significant differences were determined by Student’s t test (two groups), and one-way ANOVA test (more than two groups). *P* < 0.05 was regarded as statistically significant.

## Results

### Clinical significance of TRIM6 in CRC

qRT-PCR was performed to compare the expression of several TRIM proteins in mucosa tissues, Stage I&II CRC tissues and Stage III&IV CRC tissues (*n* = 12 per group). TRIM4, TRIM6 and TRIM11 showed significant difference between mucosa tissues and Stage I&II CRC tissues, between mucosa tissues and Stage III&IV tissues, and between Stage I&II CRC tissues and Stage III&IV tissues (Additional file 1: Fig. S1). Previous reports have demonstrated the correlation of TRIM4 [13] and TRIM11 [14] with colorectal carcinogenesis. Therefore, we focused on TRIM6 in this study.

**Fig. 1:**
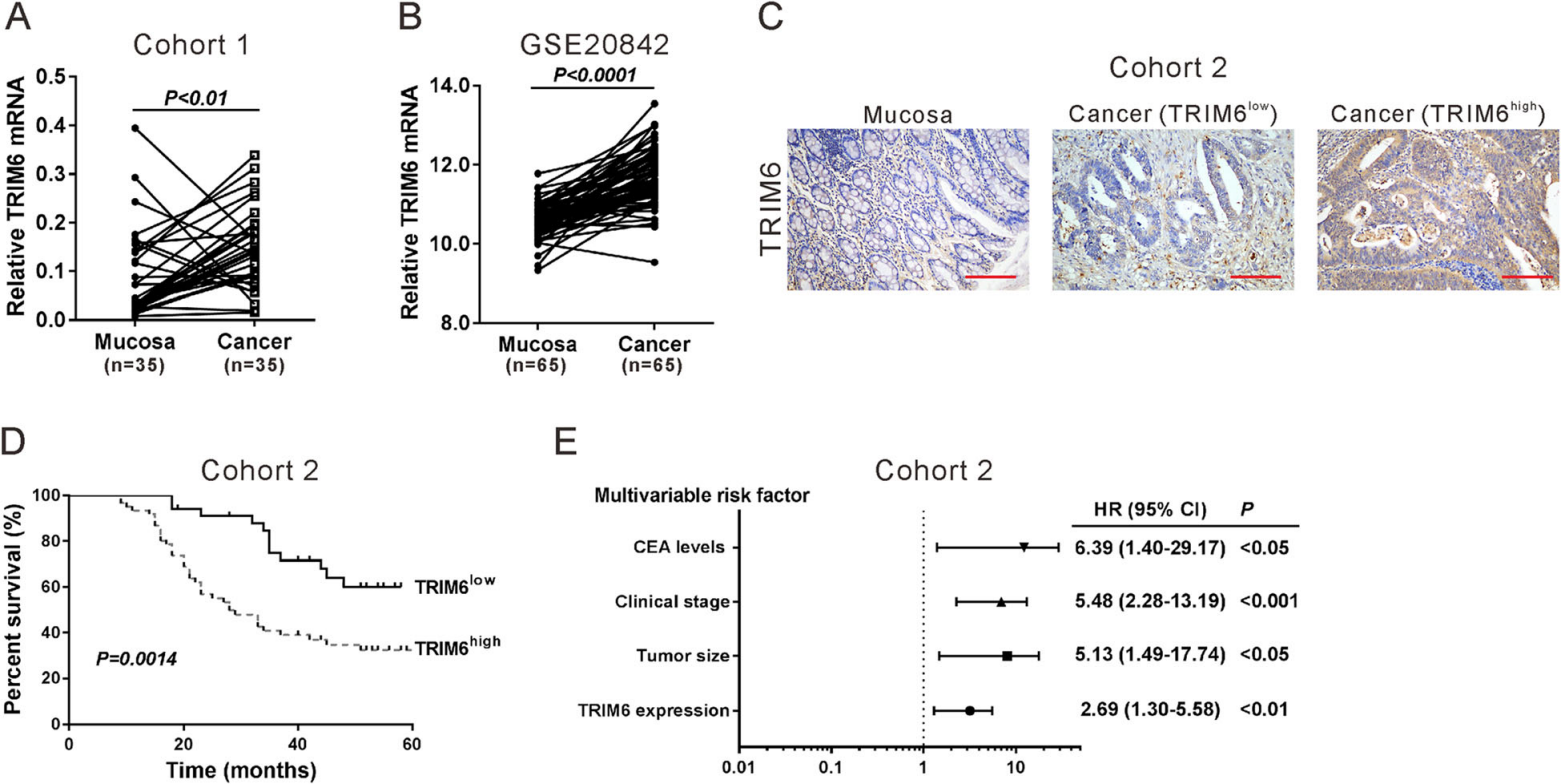
Clinical significance of TRIM6 in CRC. **a**, The mRNA expression of TRIM6 was detected in 35 pairs of CRC samples and mucosa tissues (cohort 1) by qRT-PCR. The TRIM6 expression was normalized to GAPDH. **b**, The mRNA expression of TRIM6 in GSE GSE20842 dataset, which includes 65 paired samples of cancer and adjacent mucosa from patients with Stage II/III rectal adenocarcinomas. **c**, Representative images of immunohistochemical staining for TRIM6 in CRC samples and mucosa tissues from cohort 2. Scale bar: 100 μm. **d**, Survival analysis of patients with high (TRIM6^high^) or low expression of TRIM6 (TRIM6^low^). **e**, Multivariate regression analysis in cohort 2

To confirm the increased expression of TRIM6 in CRC, qRT-PCR analysis was performed on fresh paired samples from 35 patients with CRC from Shanghai Jiao Tong University Affiliated Sixth People’s Hospital (cohort 1). As shown in Fig. 1a, TRIM6 mRNA level was elevated in cancer samples compared to that of adjacent mucosa samples (paired student’s t-test, *P* < 0.01). Consistent results were obtained with GSE20842 dataset [15], which includes 65 paired samples of tumor and adjacent mucosa from patients with Stage II/III rectal adenocarcinomas (Fig. 1b, paired student’s t-test, *P* < 0.0001).

To further explore the clinical significance of TRIM6 in CRC, IHC staining was carried out on 90 paraffin-embedded CRC samples (cohort 2). As illustrated in Fig. 1c, TRIM6 was low-expressed in normal mucosa sample, and highly expressed in 62.2% of CRC tissues (> 25% positively stained) (Table 1). Kaplan-Meier analysis and log-rank test showed that CRC patients with relative lower TRIM6 levels had better overall survival (Fig. 1d, *P* < 0.01). Fisher’s exact test was used to analyze the correlation between TRIM6 protein levels and pathological characteristics, and we found that TRIM6 levels were markedly correlated with tumor size, clinical stage, vital status and carcinoembryonic antigen (CEA) level (Table 2). In addition, multivariate Cox regression analysis revealed that TRIM6 expression was an independent prognostic marker for CRC (Fig. 1e, P < 0.01). Collectively, these results suggested that TRIM6 was upregulated in clinical CRC specimens and strongly correlated with poor prognosis.

**Table 2:** Correlation of TRIM6 expression in colorectal cancer tissues with different clinicopathological features (n = 90)

Characteristic	TRIM6		*P*-value
	Low (*n* = 34)	High (*n* = 56)	
Gender			
Male	17	31	0.6671
Female	17	25	
Age (years)			
≥ 65	12	17	0.6671
< 65	22	39	
Tumor size (cm)			
< 4.0	18	12	0.0029**
≥ 4.0	16	44	
Pathologic differentiation			
Well/Moderate	30	45	0.3943
Poor	4	11	
Clinical stage			
I/II	22	21	0.0167*
III	12	35	
Metastasis			
no	27	46	0.7857
yes	7	10	
Vital status (at followed-up)			
Alive	23	19	0.0024**
Dead	11	37	
CEA levels (ng/mL)			
< 3.5	18	14	0.0119*
≥ 3.5	16	42	

Clinicopathological features were assessed using the Fisher’s exact test. *P < 0.05, **P < 0.01

### TRIM6 knockdown inhibited CRC cell proliferation and induced G2/M arrest

**Fig. 2:**
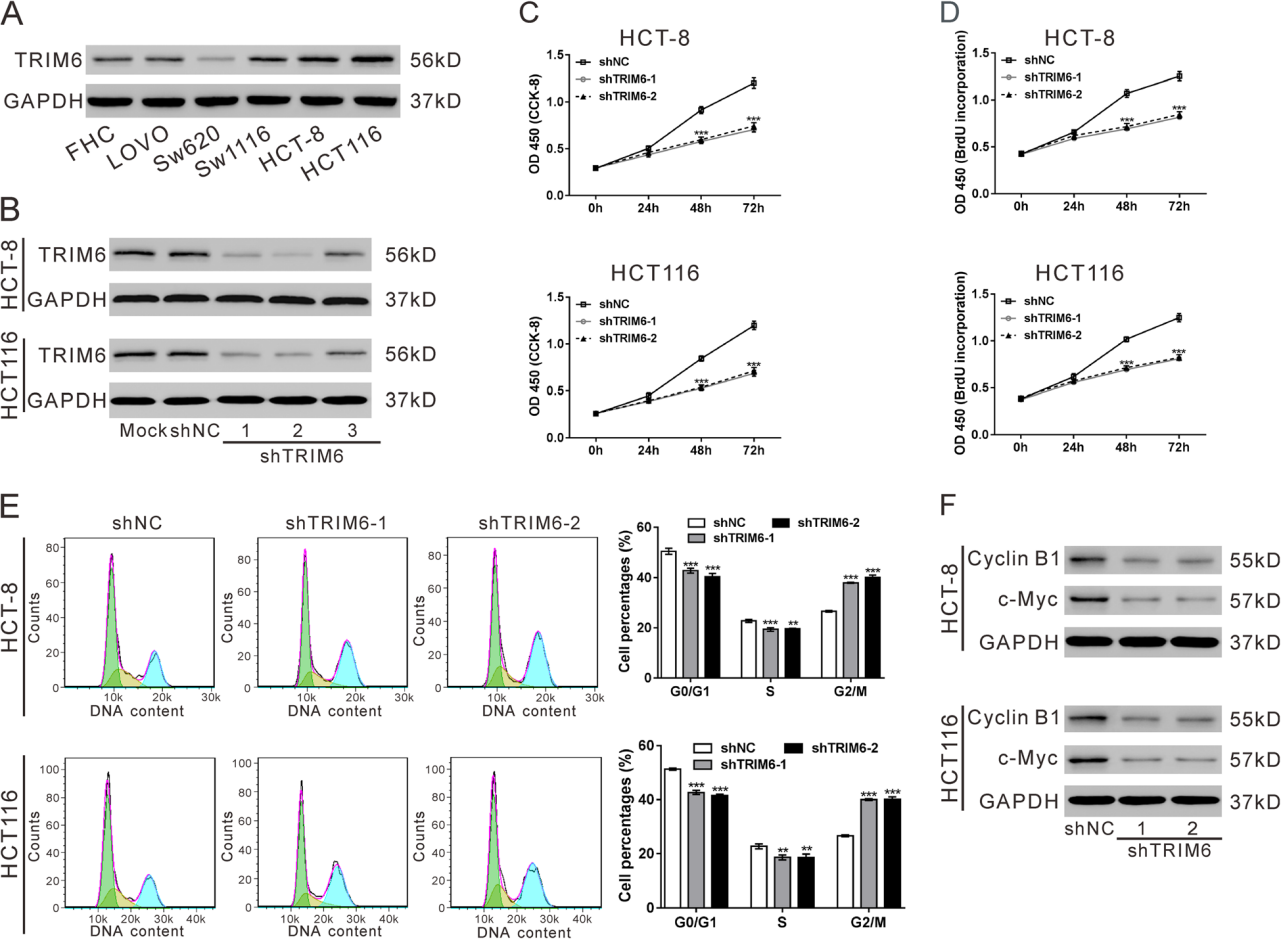
TRIM6 knockdown inhibited CRC cell proliferation and induced G2/M arrest. **a**, The protein level of TRIM6 in human normal colorectal mucosa cell line (FHC) and CRC cell lines (LOVO, Sw620, Sw1116, HCT-8 and HCT116) was examined by western blotting. GAPDH was used as a loading control. **b**, Knocking down of TRIM6 in HCT-8 and HCT116 cells. Cells were infected with lentivirus expressing TRIM6 shRNA (shTRIM6–1, − 2 and − 3) or control shRNA (shNC). At 48 h post infection, protein was extracted and TRIM6 expression was examined by western blotting. C-F, The effects of TRIM6 on the proliferation, cell cycle distribution as well as expression of Cyclin B1 and c-Myc in HCT-8 and HCT116 cells were measured by CCK-8 (**c**), BrdU (**d**), flow cytometry analyses (**e**), and western blotting (**f**), respectively. *P < 0.05, **P < 0.01, ***P < 0.001 vs shNC

To study the potential functions of TRIM6 in CRC, we first examined its expression in human normal colorectal mucosa cell line (FHC) and CRC cell lines (LOVO, Sw620, Sw1116, HCT-8 and HCT116) by western blotting (Fig. 2a). The protein levels of TRIM6 were elevated in CRC cells compared with FHC. Then, TRIM6 expression was knocked down by lentivirus-mediated TRIM6 shRNA in two cell lines with the highest TRIM6 (HCT-8 and HCT116). Western blotting results showed that shTRIM6–1 and shTRIM6–2 were efficiently decreased TRIM6 expression (Fig. 2b). Considering that the expression of TRIM6 was significant associated with tumor size, we examined the effect of TRIM6 on cell growth by CCK-8 (Fig. 2c) and BrdU assays (Fig. 2d), and the results demonstrated that downregulation of TRIM6 remarkably decreased cell proliferation in both HCT-8 and HCT116 cells. Moreover, RNAi resistant mutant of TRIM6 could rescue proliferation inhibition caused by TRIM6 shRNA (Additional file 1: Fig. S2). Cell cycle distribution was also measured by flow cytometry analysis (Fig. 2e). CRC cells with TRIM6 knockdown exhibited a significant G2/M arrest. The protein levels of cell-proliferation associated molecules, Cyclin B1 and c-Myc, were notably reduced when TRIM6 expression was knocked down (Fig. 2f). These results indicated that downregulation of TRIM6 in CRC cells repressed cell proliferation and arrested cells at G2/M phase.

### TRIM6 knockdown potentiated the anti-proliferative effects of 5-fluorouracil and oxaliplatin

**Fig. 3:**
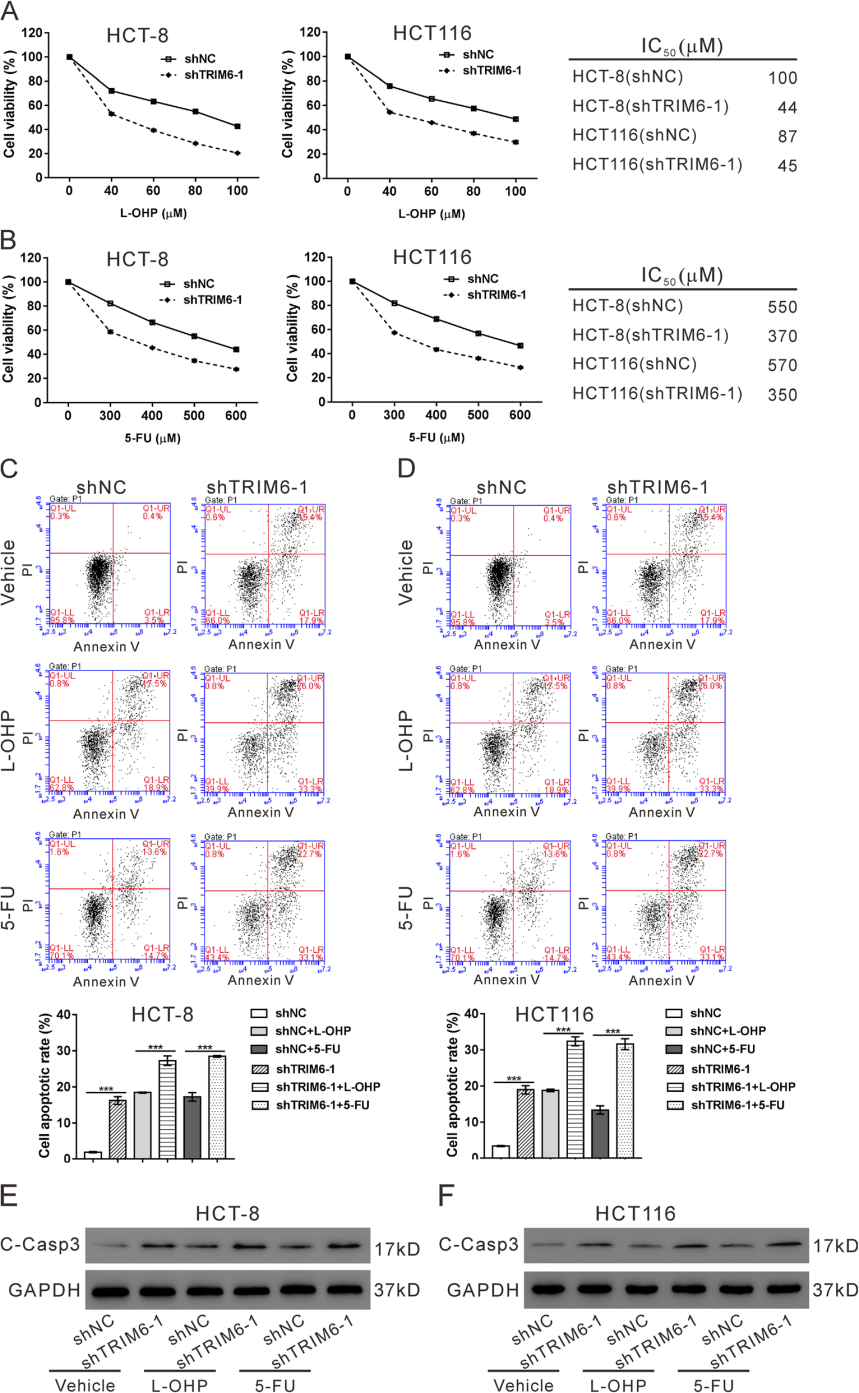
TRIM6 knockdown potentiated the anti-proliferative effects of 5-fluorouracil and oxaliplatin **a**, HCT-8 and HCT116 cells were infected with shTRIM6–1 or shNC, and treated with 40, 60, 80 or 100 μM L-OHP for 24 h. Cell proliferation was determined by CCK-8 assay, and IC50 was calculated. **b**, HCT-8 and HCT116 cells were infected with shTRIM6–1 or shNC, and treated with 300, 400, 500 or 600 μM 5-FU for 24 h. Cell proliferation was determined by CCK-8 assay, and IC50 was calculated. C-F, HCT-8 and HCT116 cells were infected with shTRIM6–1 or shNC, and treated with 64 μM L-OHP, 400 μM 5-FU or vehicle (DMSO) for 24 h. Cell apoptosis (**c**, **d**) and expression of cleaved-caspase3 (C-Casp3, **e**, **f**) was determined. ***P < 0.001

Evidence has linked TRIM proteins to chemoresistance [3–5]. 5-fluorouracil (5-FU) and oxaliplatin (L-OHP) have been widely applied to improve the outcome of CRC patients [16]. We then explored the effects of TRIM6 on 5-FU and L-OHP-treated CRC cells. As shown in Fig. 3a and b, TRIM6 knockdown markedly reduced the concentrations of 5-FU and L-OHP for achieving 50% growth inhibition in both HCT-8 and HCT116 cells. 5-FU and L-OHP treatment significantly induced the apoptosis (Fig. 3c-d) and the expression of apoptotic marker cleaved-caspase3 (C-Casp3, Fig. 3e-f) in HCT-8and HCT116 cells, which was strengthened by TRIM6 knockdown.

### TRIM6 interacted with TIS21 and promoted TIS21 ubiquitination in CRC cells

**Fig. 4:**
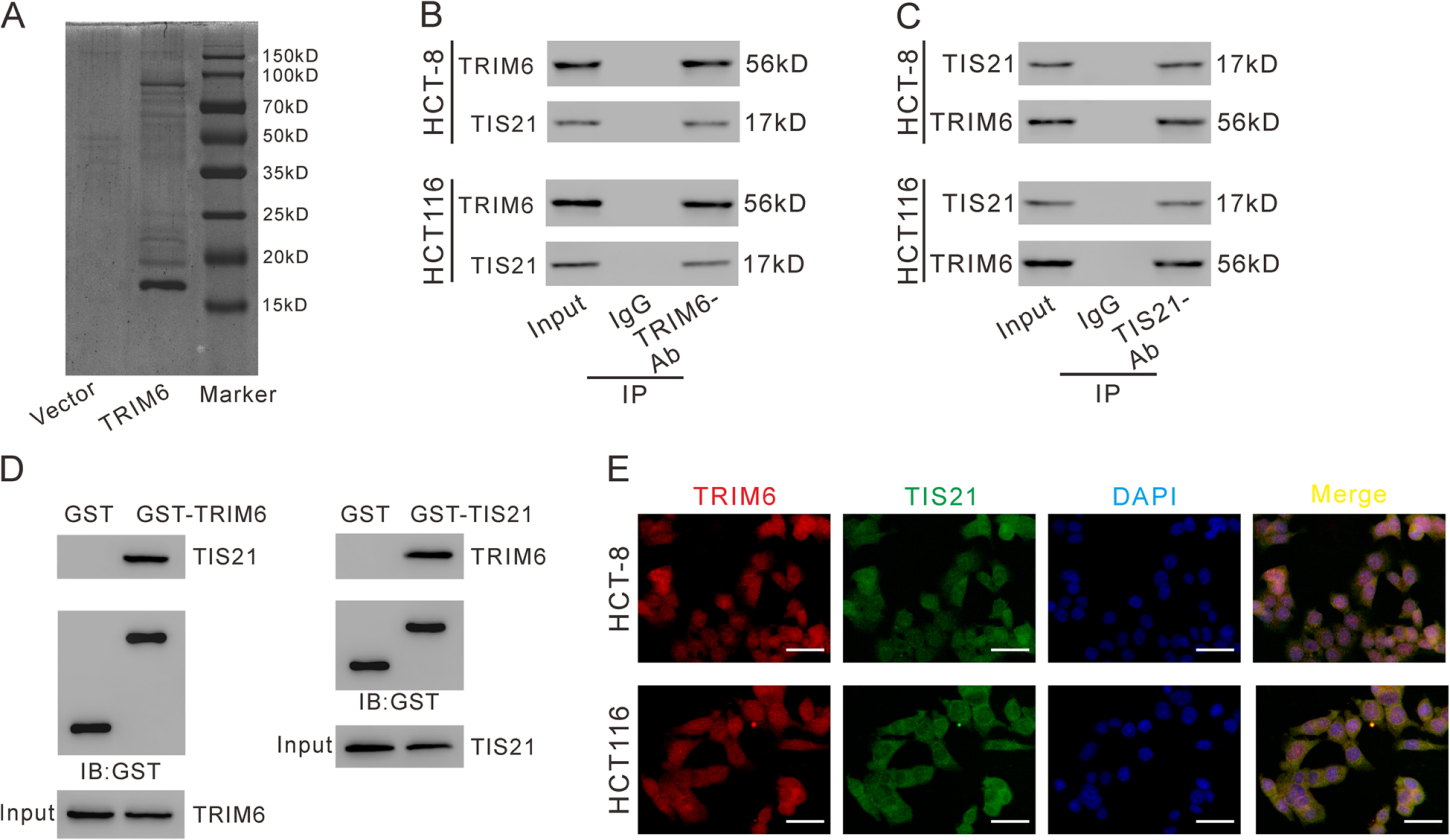
TRIM6 interacted with TIS21 in CRC cells. **a**, pCMV-Tag2-TIRM6 or pCMV-Tag2 vector was transfected into 293 T cells, and 48 h later, cell lysates were prepared and subjected to immunoprecipitation (IP) experiments with anti-FLAG beads. After elusion with FLAG peptide, the immunoprecipitated protein complexes were resolved on SDS-PAGE, and stained with Coomassie Brilliant Blue. B, C, IP was carried out with TRIM6 antibody (TRIM6-Ab) /IgG (**b**) or TIS21 antibody (TIS21-Ab) /control IgG (**c**), and then western blotting was performed to analyze specific associations between TRIM6 and TIS21 in HCT-8 and HCT116 cells. D-E, GST pull-down assay. HCT-8 cells were lysed and incubated with GST, GST-tagged TRIM6 (**d**) and GST-tagged TIS21 (**e**) bound to glutathione beads, respectively. Proteins were detected as indicated. E, immunofluorescence staining of TRIM6 (Red) and TIS21 (Green) in HCT-8 and HCT116 cells. DAPI (blue) was used to label nuclei. Scale bar: 50 μm

To investigate how TRIM6 functions in CRC, we identified candidate proteins associated with TRIM6 by Co-IP assay and proteomics analysis. Lysates from 293 T cells expressing FLAG-TRIM6 or Vector were IP with anti-FLAG beads, separated by SDS-PAGE, and stained with Coomassie Brilliant Blue. Differential expressed bands were excised (Fig. 4a) and identified by LC/MS. The results showed that 43 proteins with ≥3 peptides identified may be associated with TRIM6 (Additional file 1: Table S3). Among these 43 proteins, TIS21, which possesses antiproliferative activity [8] in a variety of human cancers, was selected for further investigation.

To validate the interaction between TRIM6 and TIS21, co-IP experiments (Fig. 4b-c), GST-pull down assay (Fig. 4d-e) and immunofluorescence staining (Fig. 4f) were performed with antibodies against TRIM6 or TIS21 in CRC cells. The results demonstrated that endogenous TRIM6 formed a complex with TIS21 in CRC cells.

**Fig. 5:**
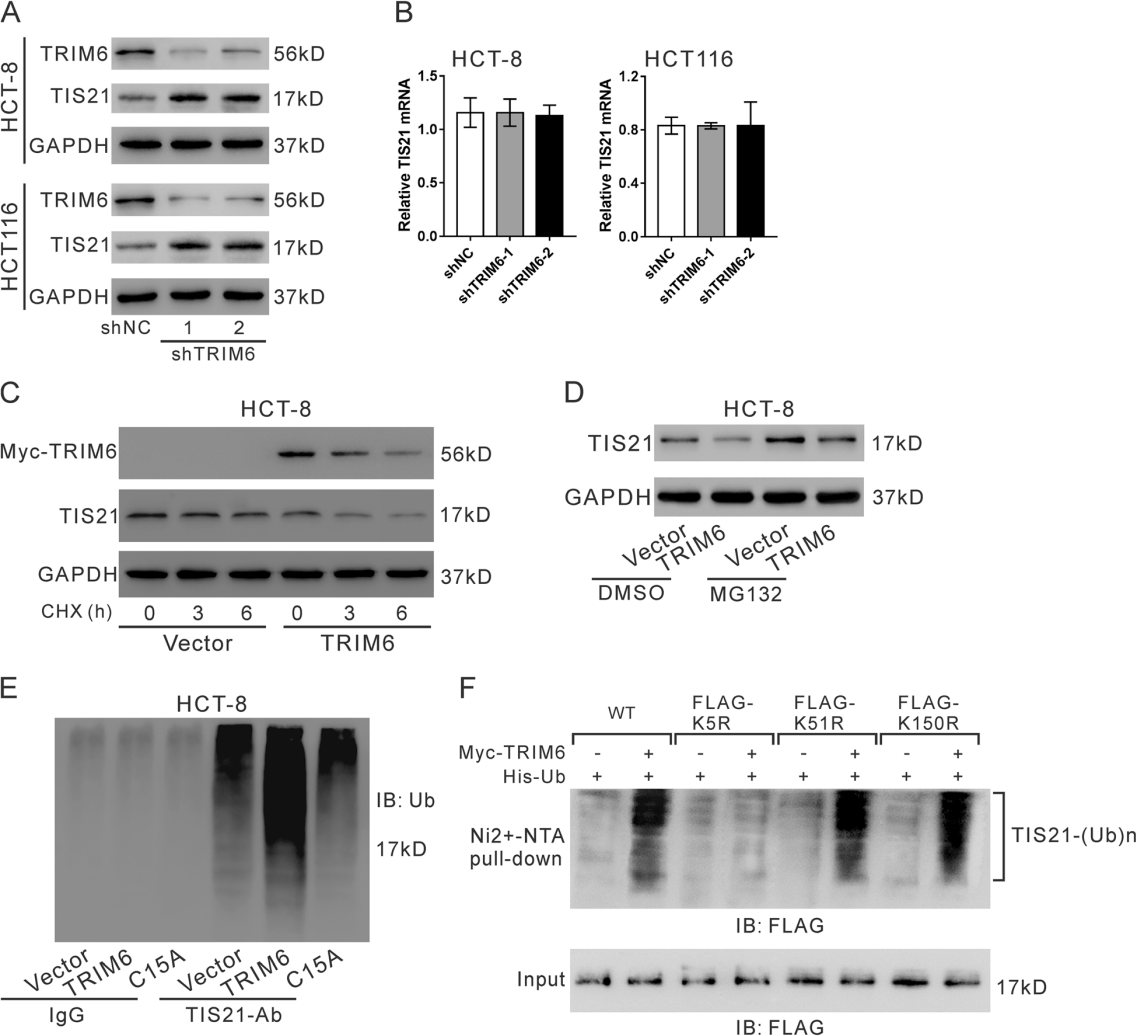
TRIM6 promoted TIS21 ubiquitination. **a**, **b**, Western blotting (**a**) and qRT-PCR (**b**) were used to detect TIS21 in HCT-8 and HCT116 cells infected with lentivirus expressing TRIM6 shRNA (shTRIM6–1, − 2) or control shRNA (shNC). **c**, HCT-8 cells were transfected with pcDNA3.1-myc-TIRM6 or pcDNA3.1-myc (Vector) for 24 h, and exposed to 20 mM cycloheximide (CHX, Sigma-Aldrich). Cell lysate was prepared at 0, 3 and 6 h after exposure and subjected to western blotting analysis. **d**, HCT-8 cells were transfected with pCMV-Tag2-TIRM6 or pCMV-Tag2 vector for 24 h and then treated with MG132 (10 μM) or DMSO for 20 h. Western blotting was used to detect TIS21. **e**, Cell lysates from HCT-8 cells infected with lentivirus expressing TRIM6 shRNA (shTRIM6–1) or control shRNA (shNC) were IP with TIS21-Ab/control IgG and then immunoblotted for ubiquitin (Ub). **f**, Ubiquitination assay. The 293 T cells were transfected with plasmids expressing myc-TRIM6, His-ubiquitin and FLAG-TIS21 (WT, K5R, K51R or K150R). Cell lysates were incubated with nickelnitrilotriacetic acid beads and subjected to western blotting with anti-FLAG

Researches had reported that TRIM6 is an E3-ubiquitin ligase [7]. It is unclear whether TRIM6 regulates TIS21 ubiquitination. Interesting, downregulation of TRIM6 enforced the expression of TIS21 protein (Fig. 5a), but had little effects on the expression of TIS2 mRNA (Fig. 5b). To determine whether TRIM6 overexpression altered TIS21 protein stability, protein synthesis was blocked by cycloheximide (CHX, 20 mM) in HCT-8 cells overexpressing Myc-tagged TRIM6. Western blotting analysis showed that TRIM6 notably decreased the half-life of TIS21 protein (Fig. 5c). To determine the involvement of proteasomal activity in TIS21 downregulation, HCT-8 cells were treated with the proteasome inhibitor MG132. As shown in Fig. 5d, MG132 treatment attenuated the reduction of TIS21 protein caused by TRIM6 overexpression. In addition, HCT-8 cells were overexpressed with wild type TRIM6 and a RING mutant TRIM6 (C15A) [8], and TIS21 ubiquitination was assessed by immunoprecipitation and Western blotting analyses. The results showed that TRIM6 overexpression significantly elevated the levels of TIS21 ubiquitination, while C15A had no such effects (Fig. 5e).

By using bio-computer analysis (http://www.ubpred.org/), three Lys residues at positions 5, 51 and 150 of TIS21 were predicted as potential ubiquitination sites. These Lys residues were then mutated to Arg and in vitro ubiquitination assay was performed. The results showed that TIS21 ubiquitination was blocked when the Lys residue at position 5 (Lys5) was replaced by Arg (Fig. 5f). Lys5 was essential for TRIM6 mediated TIS21 ubiquitination.

### TIS21/FoxM1 was essential for TRIM6-inhibited CRC cell proliferation and cell cycle progression

**Fig. 6:**
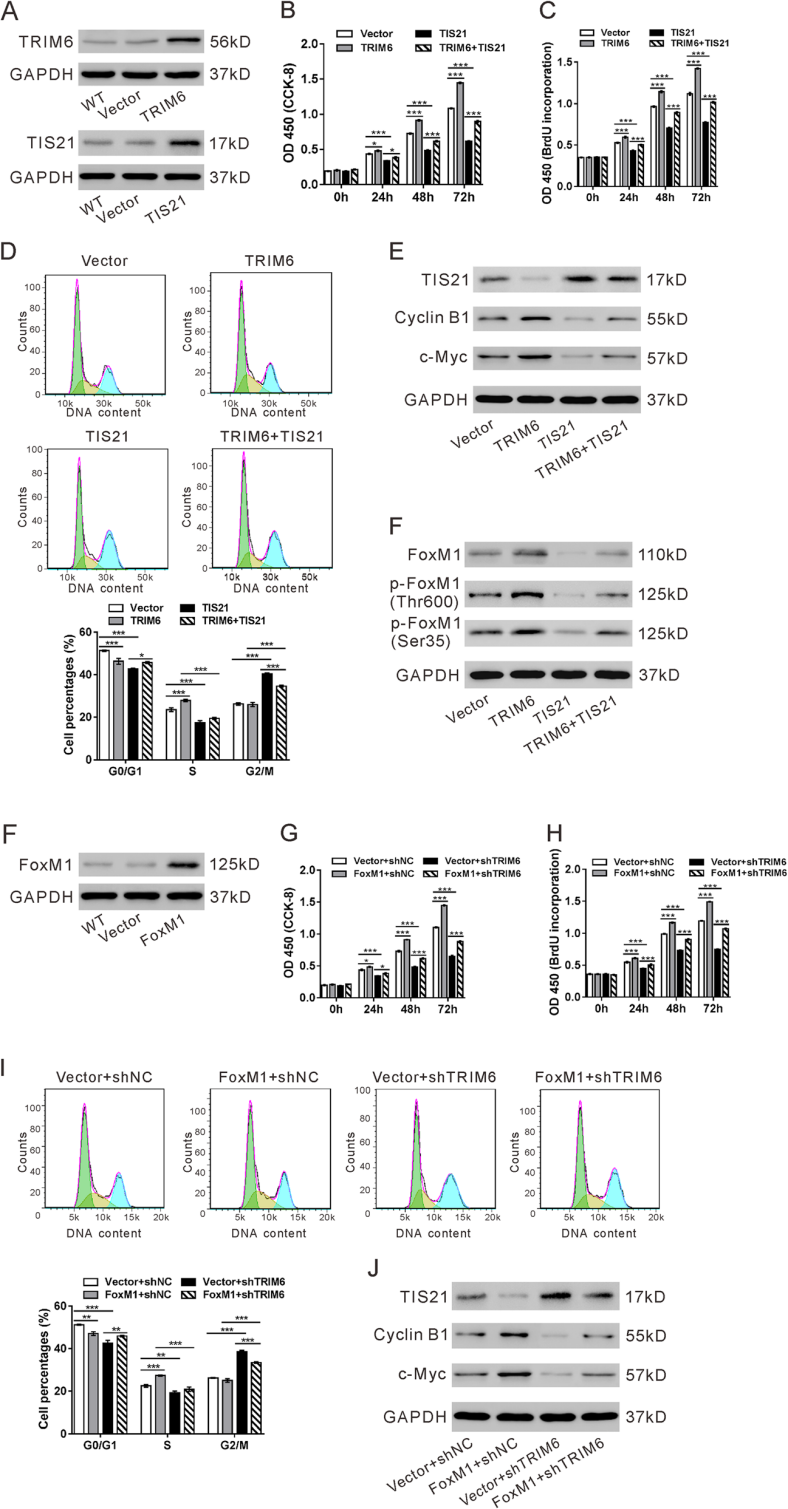
TIS21/FoxM1 was essential for TRIM6-inhibited CRC cell proliferation and cell cycle progression. **a**, Sw620 cells were transfected with plasmids expressing TRIM6, TIS21 or vector. Overexpression of TRIM6 or TIS21 was confirmed by western blotting. B-F, Sw620 cells were divided into four groups: Vector (cells transfected with vector), TRIM6 (cells transfected with plasmid expressing TRIM6), TIS21 (cells transfected with plasmid expressing TIS21) and TRIM6 + TIS21 (cells transfected with plasmid expressing TRIM6 and plasmid expressing TIS21). CCK-8 (**b**), BrdU (**c**), flow cytometry analyses (**d**) and western blotting (**e**, **f**) were performed to determine the effects of TRIM6 and TIS21 on the proliferation, cell cycle distribution and relative protein expression, respectively. F, HCT-8 cells were transfected with plasmids expressing FoxM1 or vector. Overexpression of TRIM6 or TIS21 was confirmed by western blotting. G-J, HCT-8 cells were divided into four groups: Vector+shNC (cells treated with vector and control shRNA), Vector+shTRIM6 (cells treated with vector and TRIM6 shRNA), FoxM1 + shNC (cells treated with plamid expressing FoxM1 and control shRNA) and FoxM1 + shTRIM6 (cells treated with plamid expressing FoxM1 and TRIM6 shRNA). CCK-8 (**g**), BrdU (**h**), flow cytometry analyses (**i**) and western blotting (**j**) were performed to determine the effects of FoxM1 overexpression and TRIM6 knockdown on the proliferation, cell cycle distribution and relative protein expression, respectively. *P < 0.05, **P < 0.05, ***P < 0.001

To investigate the functional roles of TIS21 in TRIM6-mediated CRC progression, we overexpressed TSI21 and TRIM6 in Sw620 cells (Fig. 6a). Overexpression of TIRM6 significantly enhanced the proliferation (Fig. 6b-c), while TIS21 overexpression rescued the promoting effect of TRIM6. In addition, CRC cells with TRIM6 overexpression had high proportions of S phase cells and low ratio of G0/G1 phase cells (Fig. 6d), and high levels of Cyclin B and c-Myc (Fig. 6e), which was also reversed by TIS21 overexpression.

It has been reported that TIS21 inhibits the activity of forkhead box M1 (FoxM1), which is a transcription factor regulating the expression of diverse mitotic genes [17]. the transcriptional activity of FoxM1 is depends on its phosphorylation level [18]. Here, TRIM6 overexpression enhanced the expression and phosphorylation of FoxM1 (Thr 600 and Ser 35), which was also blocked by TIS21 overexpression (Fig. 6f). Further, we overexpressed FoxM1 in HCT-8 cells with TRIM6 silenced. Overexpression of FoxM1 significantly reversed the effects of TRIM6 knockdown on cell proliferation (Fig. 6g-h), cell cycle distribution (Fig. 6i), and the expression of Cyclin B and c-Myc (Fig. 6j). Taken together, these results demonstrated that TRIM6 regulates proliferation and cell cycle progression in CRC cells via regulating TIS21/FoxM1.

### TRIM6 knockdown inhibited tumorigenesis of CRC cells

**Fig. 7:**
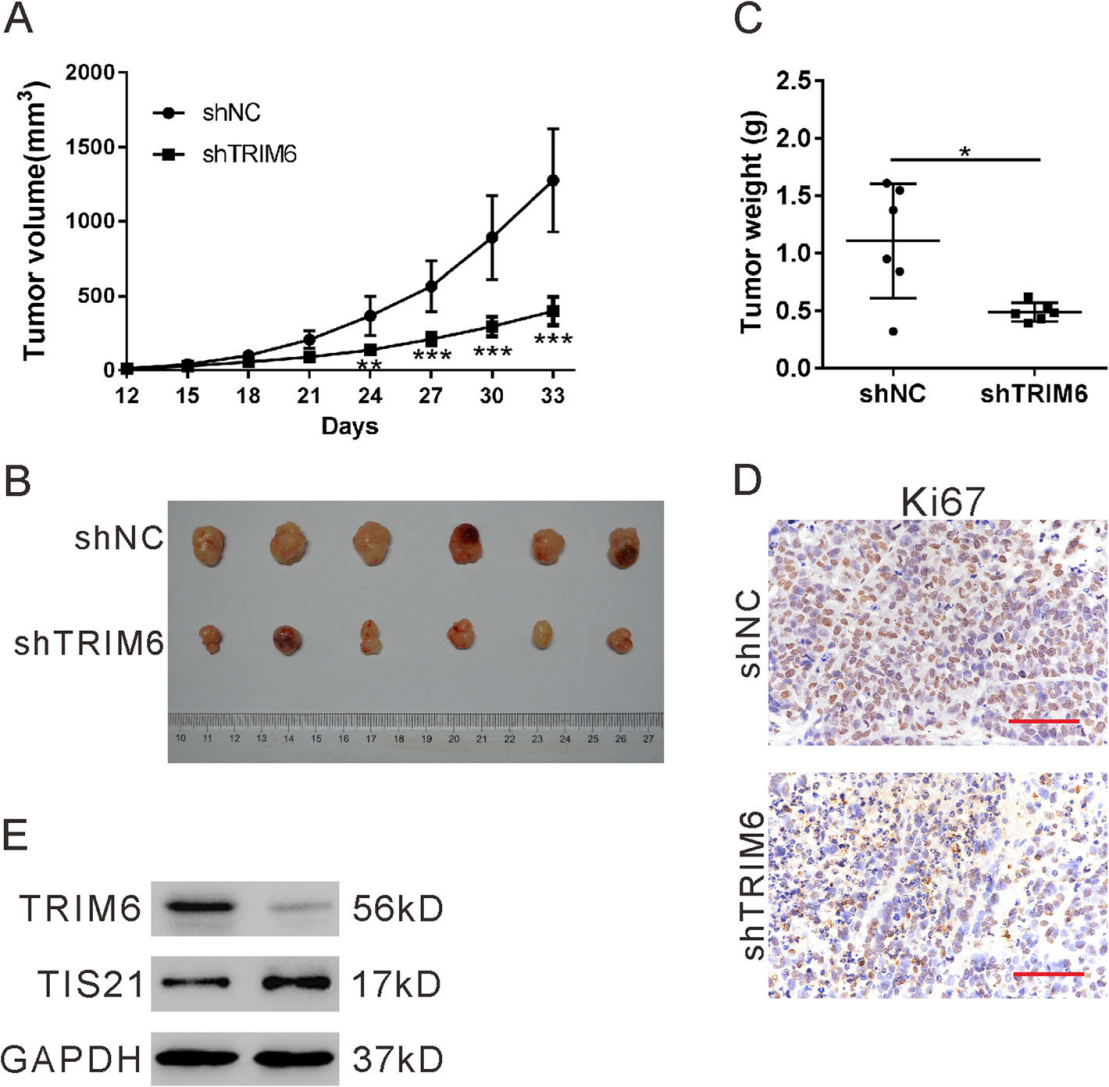
TRIM6 knockdown inhibited tumorigenesis of CRC cells. HCT-8 cells stably expressed TRIM6 shRNA (shTRIM6) or control shRNA (shNC) were injected into nude mice. Xenograft growth curve (**a**), photograph of the xenografts (**b**), tumor weight (**c**), representative images of Ki67 staining (**d**), and representative western blot (**e**) are shown. Scale bar: 50 μm. *P < 0.05, **P < 0.05, ***P < 0.001

To investigate the effect of TRIM6 knockdown on tumorigenesis, xenograft experiments were then performed by transplantation of HCT-8 cells stably expressed shTRIM6–1 or shNC into nude mice. Knocking down of TRIM6 expression in HCT-8 cells (shTRIM6–1) significantly suppressed tumor growth compared with the control cells (shNC) (Fig. 7a). At 33 days post transplantation, the size (Fig. 7b), weight (Fig. 7c), and Ki67 expression (Fig. 7d) was decreased in xenografts from shTRIM6–1 in comparison with that in shNC. In addition, xenografts from shTRIM6–1 had markedly increased protein level of TIS21 compared with shNC (Fig. 7e). Taken together, these results indicate that TRIM6 knockdown suppressed xenograft tumor growth of CRC cells.

### Correlation analyses in colorectal tissues

**Fig. 8:**
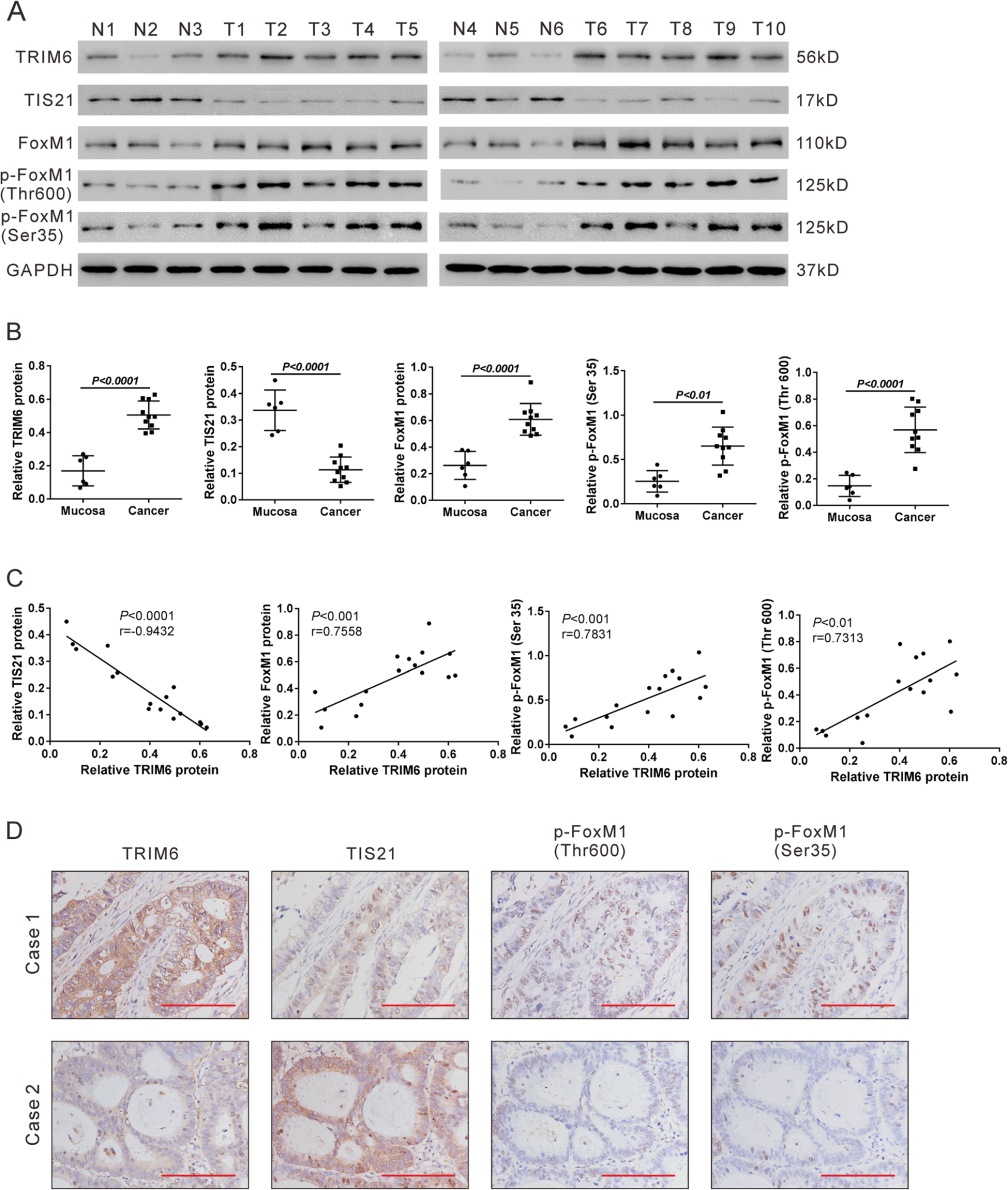
Correlation analyses in colorectal tissues. **a**, Western blotting analysis of TRIM6, TIS21, FoxM1, p-FoxM1 (Thr 600) and p-FoxM1 (Ser 35) in 6 normal mucosa samples and 10 CRC samples. **b**, Quantification of the western blotting data. **c**, Pearson correlation scatter plots in colorectal tissues. **d**, Representative images of IHC staining of TRIM6, TIS21, p-FoxM1 (Thr 600) and p-FoxM1 (Ser 35) in CRC samples (Case 1 and Case 2). Scale bar: 100 μm

**Fig. 9:**
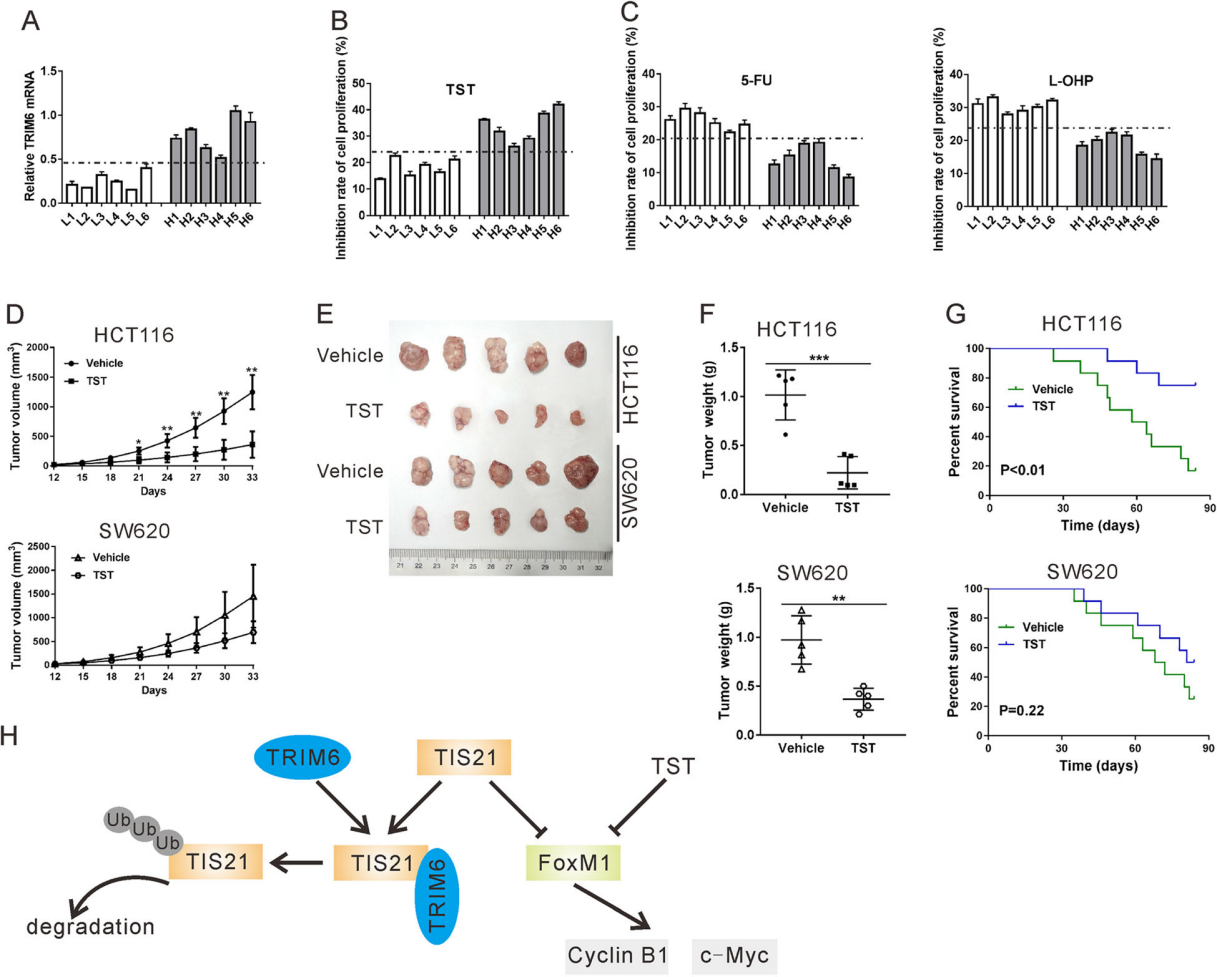
TRIM6 expression level influenced the anti-proliferative effect of FoxM1 inhibitor TST. A-C, qRT-PCR (A) was performed to detect TRIM6 mRNA expression in primary CRC cells. Cells L1-L6, had a relative lower level of TRIM6 expression than cells H1-H8. Primary CRC cells were treated with 400 μM 5-FU, 64 μM L-OHP, 2 μM TST or vehicle (DMSO) for 48 h. CCK-8 assay was carried out to determine the inhibition rate of cell proliferation (%). D-G, a xenograft mouse model was established by inoculation of HCT116 or SW620 cells to nude mice. On the 12th day after inoculation, the mice were treated with TST or Vehicle (DMSO). Tumor volume (D), tumor images (E), tumor weight (F) and overall survival (G) were shown. *P < 0.05, **P < 0.01, ***P < 0.001 vs. Vehicle. H, Schematic representation of the regulation of CRC cell proliferation by TRIM6/TIS21/FoxM1

The protein expression of TRIM6, TIS21, FoxM1, p-FoxM1 (Thr 600) and p-FoxM1 (Ser 35) was assessed in 6 normal mucosa samples and 10 CRC samples from cohort 1 by western blotting. TRIM6, FoxM1, p-FoxM1 (Thr 600) and p-FoxM1 (Ser 35) was significantly increased in CRC samples in comparison with normal mucosa samples, while TIS21 was remarkably decreased in CRC samples (Fig. 8a-b). Pearson correlation analysis (Fig. 9c) showed that TRIM6 protein expression was negatively correlated with TIS21 protein expression in colorectal tissues, whereas there was a positive correlation with FoxM1, p-FoxM1 (Thr 600) and p-FoxM1 (Ser 35). IHC staining results (Fig. 9d) also confirmed the correlation between TRIM6 and its downstream molecules, TIS21, p-FoxM1 (Thr 600) and p-FoxM1 (Ser 35). These clinical data further supported the above findings with CRC cells.

### TRIM6 expression level influenced the anti-proliferative effect of FoxM1 inhibitor TST

In vitro and in vivo experiments have supported the anti-cancer activity of FoxM1 inhibitor thiostrepton (TST) [19–22]. Given the positive correlation between TRIM6 and FoxM1 in CRC, we present a hypothesis that TRIM6 expression level influences the effect of TST on CRC cells. To find evidence supporting our hypothesis, we isolated primary CRC cells from 12 patients and these cell lines were then exposed to 5-FU, L-OHP, TST or vehicle for 48 h. As shown in Fig. 9a, these cell lines were divided into TRIM6-low expression (L1-L6) group and TRIM6-high expression (H1-H6) group. CRC cells with higher expression levels of TRIM6 were more sensitive to TST exposure (Fig. 9b), but less sensitive to 5-FU and L-OHP (Fig. 9c).

Further, a xenograft mouse model was established with HCT116 and SW620 cells and then treated with TST. As shown in Fig. 9d-g, TST treatment was effectively decreased tumor growth rate, xenograft size and xenograft weight, and improved overall survival in the mice transplanted with HCT116 cells. In the mice transplanted with SW620 cells, TST treatment was less effective. Therefore, these results suggest that the anti-cancer activity of TST in CRC is more efficient when TRIM6 expression level is higher.

## Discussion

TRIM proteins are well known to involve in immune responses and carcinogenesis [23]. The current study demonstrated the upregulated expression of TRIM6 in CRC tissues and suggested that TRIM6 expression may be an independent prognostic marker for CRC (Fig. 1). In vitro (Fig. 2 and Fig. 6) and in vivo functional experiments (Fig. 7) demonstrated that TRIM6 promoted cell cycle progression and proliferation of CRC cells. Taken together, the current study implies that TRIM6 plays oncogenic role in CRC development.

Next, we tried to explore the mechanisms by which TRIM6 influences CRC progression. We identified TIS21 as a candidate associated protein of TRIM6. Our data suggest that TRIM6 overexpression decreased TIS21 stability and increased TIS21 ubiquitination, which ascribed to the E3-ubiquitin ligase activity of TRIM6 (Fig. 5). TIS21 functions as an anti-proliferative transcriptional cofactor in fibroblast cells (NIH3T3), mouse embryo fibroblasts, breast cancer cells, prostate cancer cells and hepatocellular carcinoma (HCC) cells [17, 24–26]. In the current study, TIS21 overexpression reversed the proliferative effects of TRIM6 overexpression in CRC cells, indicating that TIS21 was a downstream regulator of TRIM6 (Fig. 6). Western blotting analysis and Pearson correlation analysis revealed a negative correlation between the protein expression of TRIM6 and TIS21 protein in clinical samples, which further validated the in vitro findings (Fig. 8).

Aberrant cell cycle activity is a main characteristic of cancer cells [27]. TRIM6 knockdown in CRC cells caused cell arrested at G2/M phase. In HCC cell line, forced expression of TIS21 significantly induced the G2/M arrest by inhibiting the activity of FoxM1 [17]. FoxM1, a forkhead box family transcription factor, involves in the regulation of DNA replication, mitosis and cell proliferation by binding to promoters of target genes [17]. In the current study, we demonstrated that TRIM6 overexpression elevated the expression and phosphorylation of FoxM1, which was blocked by TIS21 overexpression. FoxM1 overexpression reversed the effects of TRIM6 knockdown on CRC cell proliferation and cell cycle progression (Fig. 6). Cyclin B1 and c-Myc, which are required for mitotic initiation, are known as target genes of FoxM1 [28, 29]. Here, TRIM6 knockdown decreased the expression of both proteins, which was partial reversed by FoxM1 overexpression (Fig. 6j). On the contrary, TRIM6 overexpression displayed the reverse effect and such effect was blocked by TIS21 overexpression (Fig. 6f). FoxM1 expression is found to be upregulated in a number of human cancers, including CRC [30]. In general, FoxM1 overexpression is closely related to high proliferation rate and late tumor stage, and may serve as a prognostic marker for numerous human cancers [30–34]. Targeting FoxM1 may be a promising strategy for cancer [35]. Consistent with the previous report [30], we found that FoxM1 protein expression was increased in CRC tissues. More importantly, TRIM6 protein expression was positively correlated with the expression and phosphorylation of FoxM1. Taken together, TRIM6 may exert oncogenic role by regulating TIS21/FoxM1 in CRC cells. Park TJ et al. reported that TIS21 had little effect on FoxM1 expression but suppressed FoxM1 activation by binding to the CDK1/Cyclin B1 complex in HCC cells [17]. Overexpression of FoxM1 in HCC cells led to a decreased protein level of TIS21 by promoting Skp2 (S phase kinase associated protein 2)-mediated TIS21 ubiquitination [36]. Their studies revealed a regulatory loop between TIS21 and FoxM1 in HCC cells. Our current study also observed the decreased protein expression of TIS21 following the overexpression of FoxM1 in CRC cells (Fig. 6j). However, our data showed that TRM6/TIS21 affected both expression and activity of FoxM1. These different results may due to the different types of cell lines. Further experiments are required to investigate the detailed mechanisms how TIS21 downregulates FoxM1 protein expression in CRC cell lines.

Chemotherapy is one of the standard treatment options for cancer patients, but chemoresistance limits its effectiveness. The mechanism of chemoresistance is far from fully understood. Other members of TRIM family proteins, such as TRIM14, TRIM24 and TRIM98 have been reported to enhance chemoresistance in certain human cancers [3–5]. Here, we found that knockdown of TRIM6 significantly enhanced the anti-proliferative effect of 5-FU and L-OHP (Fig. 3), and primary CRC cells with higher level of TRIM6 was more resistant to 5-FU and L-OHP (Fig. 9c). Targeting FoxM1 may have promising therapeutic benefits for cancer treatment [35]. Thiostrepton (TST), a specific inhibitor for FoxM1, shows anti-cancer activity in many human cancers [19–22]. In the current study, primary CRC cells with higher level of TRIM6 was more sensitive to TST (Fig. 9c). TST had better efficacy in treating xenografts from HCT116 cells, which displayed higher level of TRIM6, than those from SW620 cells, which showed lower level of TRIM6 (Fig. 9d-g). Collectively, these data indicate that TRIM6 expression levels influence the anti-cancer efficacy of different drugs, which may be taken into account before these drugs were applied to CRC patients.

## Conclusions

The current study shows the potential prognostic value of TRIM6 in CRC and provides the first evidence that TRIM6 exerts an oncogenic role in human CRC. Our data indicate that TRIM6 may promote TIS21 ubiquitination, thus suppressing the inhibitory effects of TIS21 on FoxM1 activity and promoting CRC cell proliferation (Fig. 9h). Therefore, TRIM6 may a novel target for the treatment of CRC in the future.

## Supplementary information

**Additional file 1: Table S1.** Primers for qRT-PCR. **Table S2.** The oligo sequences for TRIM6 shRNA. **Table S3.** TRIM6 interaction proteins identified by LC/MS analysis. **Fig. S1.** The mRNA expression of several TRIM proteins was detected in mucosa tissues (*n* = 12), Stage I&II CRC tissues (n = 12) and Stage III&IV CRC tissues (n = 12) by qRT-PCR. **Fig. S2.** RNAi resistant mutant of TRIM6 rescued proliferation inhibition caused by TRIM6 shRNA in CRC cells. HCT-8 and HCT116 cells were infected with lentivirus expressing TRIM6 shRNA (shTRIM6–1 and − 2) or control shRNA (shNC), and transfected with plasmids expressing RNAi resistant mutant TRIM6. A, TRIM6 expression was detected by western blotting. B, Cell proliferation was assessed by CCK-8 assay. **P < 0.01.

## Data Availability

The data and materials of this study are available from the corresponding authors for reasonable requests.

## Abbreviations

BrdU: Bromodeoxyuridine
CCK-8: Cell counting kit-8
CRC: colorectal cancer
IHC: immunohistochemica
IKKε: IκB kinase-ε
IP: Immunoprecipitation
ISGs: IFN-I stimulated genes
LC/MS: Liquid chromatography/mass spectrometry
qRT-PCR: Quantitative RT-PCR
TRIM: Tripartite motif-containing proteins
TST: Thiostrepton
