# Correction: TRIM6 promotes colorectal cancer cells proliferation and response to thiostrepton by TIS21/FoxM1

**DOI:** 10.1186/s13046-022-02434-x

**Published:** 2022-07-13

**Authors:** Shuier Zheng, Chenliang Zhou, Yonggang Wang, Hongtao Li, Yong Sun, Zan Shen

**Affiliations:** Department of Oncology, Shanghai Jiao Tong University Affiliated Sixth People’s Hospital, 600 Yishan Road, Shanghai, 200233 China

**Correction: J Exp Clin Cancer Res 39, 23 (2020)**

**https://doi.org/10.1186/s13046-019-1504-5**

Following publication of the original article [1], an error was identified in Fig. 3; specifically:

- Figure 3C: Flow of cytometry pictures are incorrect; correct image is now used

**Fig. 3:**
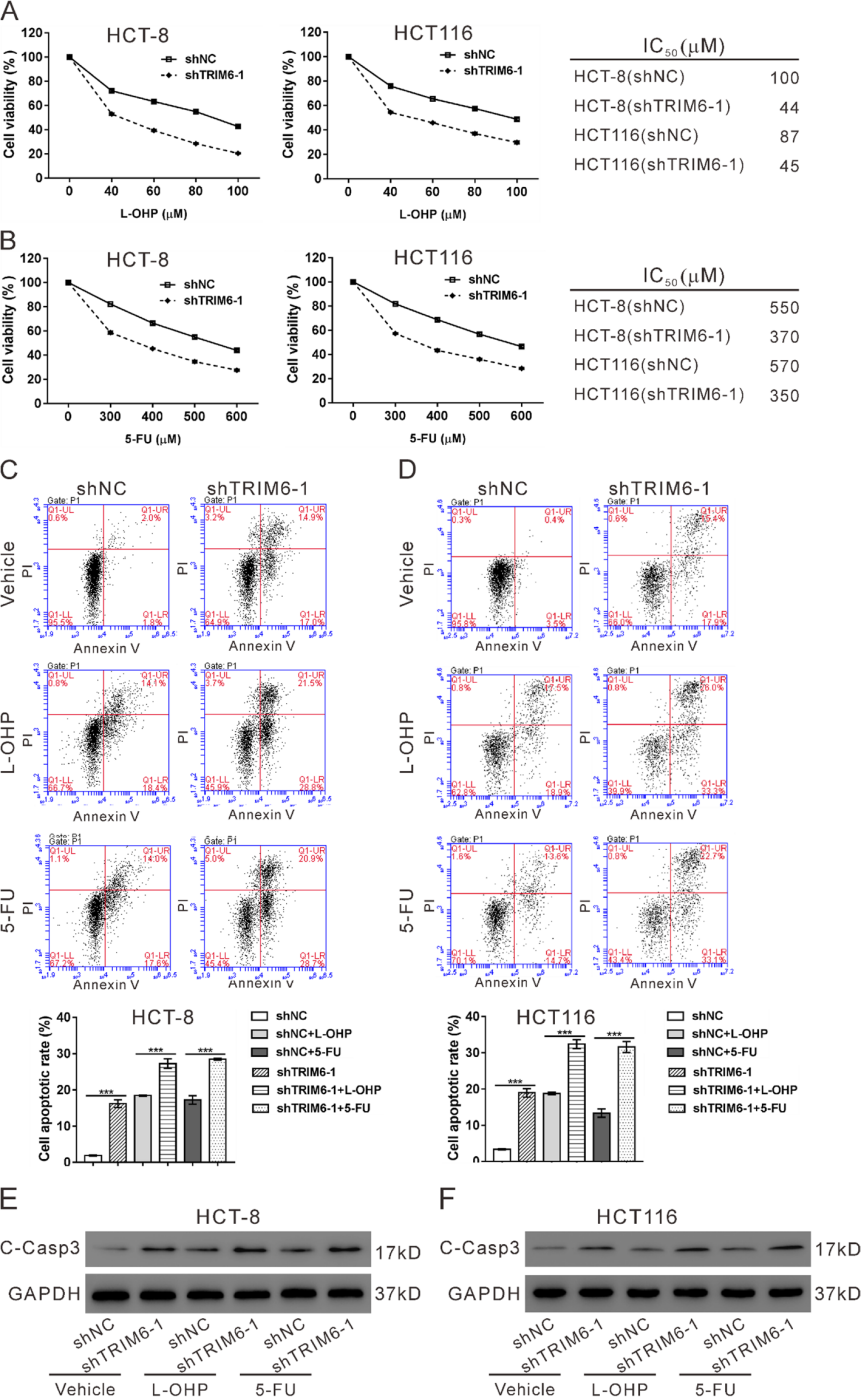
TRIM6 knockdown potentiated the anti-proliferative effects of 5-fluorouracil and oxaliplatin **a**, HCT-8 and HCT116 cells were infected with shTRIM6–1 or shNC, and treated with 40, 60, 80 or 100 μM L-OHP for 24 h. Cell proliferation was determined by CCK-8 assay, and IC50 was calculated. **b**, HCT-8 and HCT116 cells were infected with shTRIM6–1 or shNC, and treated with 300, 400, 500 or 600 μM 5-FU for 24 h. Cell proliferation was determined by CCK-8 assay, and IC50 was calculated. C-F, HCT-8 and HCT116 cells were infected with shTRIM6–1 or shNC, and treated with 64 μM L-OHP, 400 μM 5-FU or vehicle (DMSO) for 24 h. Cell apoptosis (**c**, **d**) and expression of cleaved-caspase3 (C-Casp3, **e**, **f**) was determined. ***P < 0.001

The correction does not have any effect on the results or conclusions of the paper.
