# Supplementary material for: TRIM6 promotes colorectal cancer cells proliferation and response to thiostrepton by TIS21/FoxM1
Source: J Exp Clin Cancer Res. 2020 Jan 28;39:23. doi: 10.1186/s13046-019-1504-5 (PMC6988281; doi:10.1186/s13046-019-1504-5)
Supplement: Supplementary file 1 — Additional file 1: Table S1. Primers for qRT-PCR. Table S2. The oligo sequences for TRIM6 shRNA. Table S3. TRIM6 interaction proteins identified by LC/MS analysis. Fig. S1. The mRNA expression of several TRIM proteins was detected in mucosa tissues (n = 12), Stage I&II CRC tissues (n = 12) and Stage III&IV CRC tissues (n = 12) by qRT-PCR. Fig. S2. RNAi resistant mutant of TRIM6 rescued proliferation inhibition caused by TRIM6 shRNA in CRC cells. HCT-8 and HCT116 cells were infected with lentivirus expressing TRIM6 shRNA (shTRIM6–1 and − 2) or control shRNA (shNC), and transfected with plasmids expressing RNAi resistant mutant TRIM6. A, TRIM6 expression was detected by western blotting. B, Cell proliferation was assessed by CCK-8 assay. **P < 0.01. [file 13046_2019_1504_MOESM1_ESM.docx]

**Table S1.** Primers for qRT-PCR.

| **Gene** | **Forward primer (5’-3’)** | **Reverse primer (5’-3’)** |
| --- | --- | --- |
| TRIM4 | ACCTCAGGGAAACATTAC | GCAGCACTCCAATAAATC |
| TRIM5 | GCCTCTGACACTGACTAAGAAG | GGTAAACTGACACAGGGCTAAG |
| TRIM6 | CTTTCCCACTACTCTTTGTC | TAAGCCTCAGGGTACTTATC |
| TRIM7 | AGGGTGTCCACATAATTGTTG | ATGCCATGAGGCTCTTTATTG |
| TRIM10 | GCTCTGTCATCTCCCTACTG | CAGGTTTGGGTGCTTTGG |
| TRIM11 | CACCTAAGCTGCACAGTTCC | GGCTGCCTCCTAATTCTTCC |
| TRIM15 | GCCCATCTTCACCTTCAC | CCCACTTCAGCCTTTCAG |
| TRIM17 | ACGGAAGAAGAGGAGACTG | AATCTGTCCGGGAACTCTG |
| TRIM22 | TCCATAGCAAAGCATCATAG | GACAATGTGAAGAGTCATAG |
| TRIM25 | GTCTCTACCCAGAACAGTTTCC | ATCCAACACAGGCTGATTCC |
| TRIM26 | GCTTCATTCCAGCCTCGCAGTC | TCACAGGGTCCCGCAGGTAATC |
| TRIM27 | TGCCATCACCCAGTTCTC | AGCCCTGCTCAATGTGTC |
| GAPDH | AATCCCATCACCATCTTC | AGGCTGTTGTCATACTTC |

**Table S2**. The oligo sequences for TRIM6 shRNA.

| shTRIM6#1 |  |
| --- | --- |
| sense | CCGGTCCGGAGACAAGTGAGGTTTCTCGAGAAACCTCACTTGTCTCCGGTTTTTG |
| antisense | AATTCAAAAACCGGAGACAAGTGAGGTTTCTCGAGAAACCTCACTTGTCTCCGGA |
| shTRIM6#2 |  |
| sense | CCGGTGCTAAAGTATCTGGACCTTCTCGAGAAGGTCCAGATACTTTAGCTTTTTG |
| antisense | AATTCAAAAAGCTAAAGTATCTGGACCTTCTCGAGAAGGTCCAGATACTTTAGCA |
| shTRIM6#3 |  |
| sense | CCGGTCCATGAATATAGGGCCTATCTCGAGATAGGCCCTATATTCATGGTTTTTG |
| antisense | AATTCAAAAACCATGAATATAGGGCCTATCTCGAGATAGGCCCTATATTCATGGA |
| shNC |  |
| sense | CCGGTCCGGAGACAAGTGAGGTTTCTCGAGAAACCTCACTTGTCTCCGGTTTTTG |
| antisense | AATTCAAAAACCGGAGACAAGTGAGGTTTCTCGAGAAACCTCACTTGTCTCCGGA |

**Table S3.** TRIM6 interaction proteins identified by LC/MS analysis.

| **Protein** | **Accession** | **Score** | **Coverage** | **# Proteins** | **# Unique Peptides** | **# Peptides** | **# PSMs** | **# AAs** | **MW [kDa]** | **calc. pI** |
| --- | --- | --- | --- | --- | --- | --- | --- | --- | --- | --- |
| TRIM6 | Q9C030 | 20876.46 | 47.98 | 2 | 62 | 62 | 1069 | 892 | 56.4 | 8.07 |
| ALBU | P02768 | 4625.42 | 36.12 | 1 | 38 | 38 | 252 | 609 | 69.3 | 6.28 |
| MYH6 | P13533 | 4131.14 | 40.02 | 5 | 29 | 88 | 207 | 1939 | 223.6 | 5.73 |
| TIS21 | P78543 | 4077.78 | 40.08 | 6 | 19 | 24 | 189 | 479 | 17.4 | 8.22 |
| MYH7 | P12883 | 3323.09 | 30.90 | 5 | 7 | 67 | 159 | 1935 | 223.0 | 5.80 |
| ACTA | P62736 | 3038.38 | 54.91 | 5 | 8 | 21 | 145 | 377 | 42.0 | 5.39 |
| ACTG2 | P63267 | 3018.50 | 35.73 | 8 | 4 | 17 | 146 | 375 | 41.9 | 5.16 |
| HSP7C | P11142 | 2218.60 | 39.78 | 2 | 25 | 30 | 139 | 646 | 70.9 | 5.52 |
| DB109 | Q30KR1 | 1924.48 | 68.97 | 1 | 3 | 3 | 45 | 87 | 9.9 | 8.53 |
| RAB1A | P62820 | 1835.17 | 32.80 | 12 | 3 | 23 | 137 | 564 | 60.0 | 8.00 |
| LCN1 | P31025 | 1607.57 | 29.83 | 11 | 13 | 23 | 138 | 590 | 19.3 | 5.30 |
| DB126 | Q9BYW3 | 1594.97 | 23.42 | 1 | 3 | 3 | 37 | 111 | 12.2 | 9.31 |
| FLNA | P21333 | 1537.22 | 27.77 | 3 | 69 | 69 | 108 | 2647 | 280.6 | 6.06 |
| TBB3 | Q13509 | 1412.96 | 17.78 | 1 | 3 | 9 | 62 | 450 | 50.4 | 4.93 |
| ANM5 | O14744 | 1401.32 | 29.67 | 1 | 26 | 26 | 124 | 637 | 72.6 | 6.29 |
| HS90B | P08238 | 286.39 | 22.93 | 4 | 8 | 16 | 25 | 724 | 83.2 | 5.03 |
| APOA1 | P02647 | 1253.74 | 76.40 | 1 | 34 | 34 | 75 | 267 | 30.8 | 5.76 |
| APOB | P04114 | 1225.70 | 14.51 | 1 | 62 | 62 | 72 | 4563 | 515.3 | 7.05 |
| CO3 | P01024 | 1178.14 | 31.81 | 2 | 52 | 52 | 79 | 1663 | 187.0 | 6.40 |
| ABCF1 | Q8NE71 | 308.26 | 26.81 | 1 | 16 | 16 | 24 | 623 | 71.2 | 7.37 |
| KIF11 | P52732 | 815.13 | 28.50 | 1 | 34 | 34 | 61 | 1056 | 119.1 | 5.64 |
| NOP56 | O00567 | 762.13 | 40.11 | 1 | 19 | 19 | 43 | 546 | 66.1 | 9.77 |
| STK38 | Q15208 | 749.91 | 32.69 | 1 | 18 | 22 | 63 | 465 | 54.2 | 7.15 |
| GRP75 | P38646 | 697.73 | 34.90 | 1 | 21 | 21 | 37 | 679 | 73.6 | 6.16 |
| HSP71 | P08107 | 693.91 | 15.29 | 2 | 6 | 10 | 36 | 641 | 70.0 | 5.66 |
| GSTP1 | P09211 | 635.78 | 58.10 | 1 | 11 | 11 | 36 | 210 | 23.3 | 5.64 |
| IF4B | P23588 | 625.69 | 33.22 | 1 | 19 | 19 | 56 | 611 | 69.1 | 5.73 |
| GTF2I | P78347 | 619.54 | 26.75 | 3 | 28 | 28 | 48 | 998 | 112.3 | 6.39 |
| RS3 | P23396 | 608.38 | 54.73 | 1 | 17 | 17 | 47 | 243 | 26.7 | 9.66 |
| KCTD2 | Q14681 | 599.94 | 22.43 | 1 | 3 | 4 | 28 | 263 | 28.5 | 5.29 |
| LMNA | P02545 | 594.41 | 23.80 | 1 | 15 | 15 | 27 | 664 | 74.1 | 7.02 |
| FIBA | P02671 | 573.70 | 28.29 | 1 | 24 | 24 | 51 | 866 | 94.9 | 6.01 |
| ATPA | P25705 | 545.01 | 34.90 | 1 | 21 | 21 | 32 | 553 | 59.7 | 9.13 |
| TCPB | P78371 | 508.20 | 26.92 | 1 | 15 | 15 | 28 | 535 | 57.5 | 6.46 |
| TBA1C | Q9BQE3 | 501.41 | 27.39 | 9 | 15 | 15 | 45 | 449 | 49.9 | 5.10 |
| ATPB | P06576 | 496.99 | 38.19 | 1 | 15 | 15 | 31 | 529 | 56.5 | 5.40 |
| EF1A1 | P68104 | 491.93 | 18.61 | 3 | 11 | 11 | 39 | 462 | 50.1 | 9.01 |
| A2MG | P01023 | 488.72 | 18.52 | 2 | 24 | 24 | 35 | 1474 | 163.2 | 6.46 |
| ADT2 | P05141 | 469.17 | 29.19 | 2 | 7 | 14 | 33 | 298 | 32.8 | 9.69 |
| HNRH1 | P31943 | 440.84 | 17.59 | 2 | 7 | 8 | 25 | 449 | 49.2 | 6.30 |
| EFTU | P49411 | 436.78 | 35.84 | 1 | 15 | 15 | 31 | 452 | 49.5 | 7.61 |
| GFPT1 | Q06210 | 401.77 | 32.33 | 2 | 20 | 20 | 28 | 699 | 78.8 | 7.11 |
| HORN | Q86YZ3 | 396.92 | 10.63 | 1 | 9 | 9 | 20 | 2850 | 282.2 | 10.04 |
| IGHG1 | P01857 | 392.41 | 29.39 | 1 | 8 | 11 | 35 | 330 | 36.1 | 8.19 |


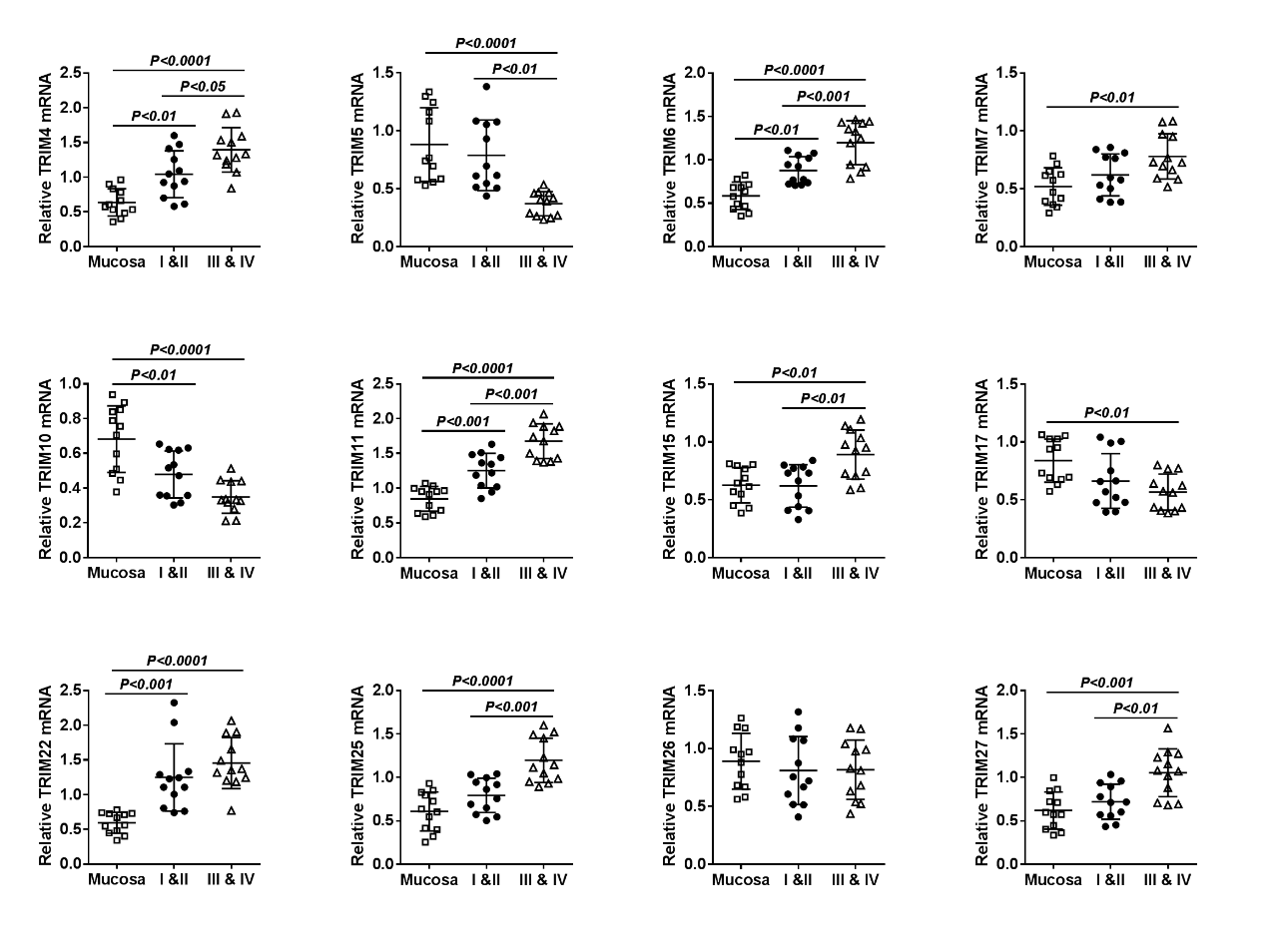


**Figure S1.** The mRNA expression of several TRIM proteins was detected in mucosa tissues (n=12), Stage I&II CRC tissues (n=12) and Stage III&IV CRC tissues (n=12) by qRT-PCR.


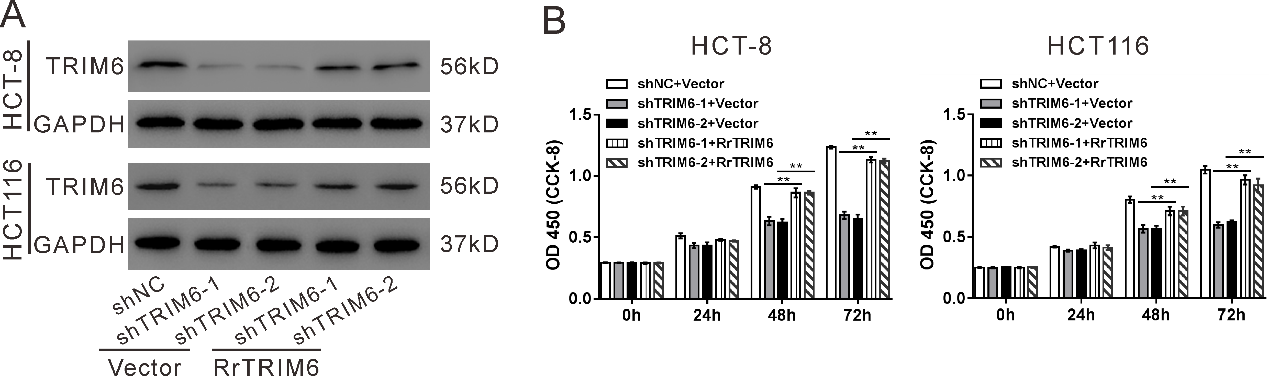


**Figure S2.** RNAi resistant mutant of TRIM6 rescued proliferation inhibition caused by TRIM6 shRNA in CRC cells. HCT-8 and HCT116 cells were infected with lentivirus expressing TRIM6 shRNA (shTRIM6-1 and -2) or control shRNA (shNC), and transfected with plasmids expressing RNAi resistant mutant TRIM6. A, TRIM6 expression was detected by western blotting. B, Cell proliferation was assessed by CCK-8 assay. **P<0.01.
